# Finding a molecular genetic marker for the incidence of recurrent episodes of acute obstructive bronchitis in children

**DOI:** 10.25122/jml-2021-0052

**Published:** 2021

**Authors:** Nataliia Lukianenko, Olena Kens, Zhansulu Nurgaliyeva, Dinara Toguzbayeva, Musa Sakhipov

**Affiliations:** 1. Department of Clinical Genetics, Institute of Hereditary Pathology of the National Academy of Medical Sciences of Ukraine, Lviv, Ukraine; 2. Department of Propaedeutics of Pediatrics and Medical Genetics, Danylo Halytsky Lviv National Medical University, Lviv, Ukraine; 3. Department of Pharmacology, West Kazakhstan Marat Ospanov State Medical University, Aktobe, Republic of Kazakhstan; 4. Department of Otorhinolaryngology, Kazakh Medical University of Continuing Education, Almaty, Republic of Kazakhstan; 5. Department of Surgery with a Course of Anesthesiology and Resuscitation, Kazakh-Russian Medical University, Almaty, Republic of Kazakhstan

**Keywords:** testing, *IL4* gene, single nucleotide polymorphism C-33T, Student criterion

## Abstract

Over the last ten years, the incidence of the pathology of the bronchus-pulmonary system in children has increased 3.6 times, mainly due to acute and recurrent inflammatory diseases of the upper and lower respiratory tract. Thus, the problem of identifying children with recurrent episodes of acute obstructive bronchitis and an increased risk of developing asthma is relevant and promising. The goal of this study was to find molecular genetic markers associated with increased susceptibility of children to repeated episodes of acute obstructive bronchitis. The molecular genetic testing of the *IL4* gene of a single nucleotide polymorphism C-33T was performed in 35 children with recurrent episodes of acute obstructive bronchitis and 35 children with acute bronchitis. The results were statistically processed on a personal computer with the calculation of values the arithmetic mean (M), of the errors arithmetic mean (m), Student criterion (t), the degree of probability (p), Pearson criterion (χ^2^), and the odds ratio (OR). Statistically significant differences were figured at p<0.01 and p<0.05. It has been proved that the presence of a child’s genotype 33CT *IL4* increases the risk of recurrent acute obstructive bronchitis four times.

## Introduction

Diseases of respiratory disease among children from 0 to 14 years represented 25.0% of the total number of pediatric consultations [1]. Over the last ten years, the incidence of the pathology of the bronchus-pulmonary system in children has increased 3.6 times, mainly due to acute and recurrent inflammatory diseases of the upper and lower respiratory tract [2, 3]. Along with the high incidence of acute respiratory infections in children, currently, there is also a high incidence of acute obstructive bronchitis, which according to various authors, occurs in 10.0–30.0% of children and greatly complicates the treatment of children as outpatients [4–6]. Many patients in this group required hospital treatment, which is neither economically nor beneficial for the state, and a stressful situation for both the child and the parents [7–9]. Uncontrolled inflammation in the bronchi with repeated episodes of acute obstructive bronchitis eventually leads to reduced lung function, physiological-pathological consequences in the form of structural damage remodeling bronchus involving the alveoli, which in turn leads to chronic obstructive pulmonary disease or asthma [2, 10–12].

According to the literature, there is no consensus on the reliable prediction of markers of asthma in a child suffering from recurrent episodes of acute obstructive bronchitis, the boundary between these diseases is quite subjective [13, 14]. Analysis of lung function and the level of nitric oxide in exhaled air is considered a high informative marker of asthma, but, unfortunately, these studies are not feasible in children under 6 years of age [15]. At this time, it is known that within each population of individuals, there are hereditary variations in the Deoxyribonucleic acid (DNA) sequence called polymorphisms, which occur in the human genome at a frequency of 1 per 1.000 base pairs. Despite this prevalence, only a small portion of these is functionally important, which leads to changes in protein products, including cytokines, thereby identifying individual responses to the damaging factors [16]. The *IL4* gene is often called “critical inflammatory cytokines”. Anti-inflammatory cytokine activates humoral immunity, controls the proliferation and differentiation of B cells and T helper cells, and increases immunoglobulin E production, which can provoke allergic reactions and airway inflammation [17]. Also, it increases the cytokinetics activity of macrophages, promotes the migration of inflammatory neutrophils, and increases the production of colony-stimulating factors. The most important functional polymorphisms of gene *IL4* are C-590T and C33T. Some researchers have shown the association of these polymorphisms with the development of atopic asthma, obstructive pulmonary disease, nasal polyposis. In this manner, the *IL4* gene is directly involved in the regulation of immune response.

That is why research on polymorphic variants of this gene is essential in children with acute respiratory infections. Implementation of these methods allows for early diagnosis, timely treatment, and prevention of acute obstructive bronchitis, which will reduce the incidence in children. The goal of this study was to find molecular genetic markers associated with increased susceptibility of children to repeated episodes of acute obstructive bronchitis.

## Material and Methods

This paper is a scientific study that focuses on finding molecular genetic markers associated with the increased tendency of children towards recurrent episodes of acute obstructive bronchitis. The participants of the study are young patients with chronic nonspecific lung diseases. The subject of the study included the epidemiology of chronic bronchitis, the features of the influence of the studied nosologies on the quality of life of young patients, and the development of a mathematical program for the diagnosis of chronic bronchitis. The theoretical framework of the study included previously published works on the problems identified in the course of acute chronic diseases in young children, studies on the identification of the propensity of children to bronchial diseases [18–21].

Upon writing the study, general scientific and special methods were used: systemic analysis is a methodology of systems theory, which comprises studying any objects represented as systems, carrying out their structuring, and subsequent analysis. The main feature of the systemic analysis is that it includes not only methods of analysis (from the Greek “analysis” – the dismemberment of an object into elements), but also synthesis methods (from the Greek “synthesis” – the combination of elements into a single whole. The main goal of systemic analysis is to detect and eliminate uncertainty in solving a complex problem based on finding the best solution from existing alternatives [15]. The systemic analysis helped analyze the stages of testing in children with an increased tendency towards recurrent episodes of acute obstructive bronchitis to identify patterns in their analyses.

The comparative analysis method is a powerful and versatile tool that enhances understanding and describes political processes and changes in the sphere under study. The features of the course of the disease were determined in children with a single case of acute obstructive bronchitis and children with recurrence and relapses of this disease, using comparative analysis. Also, the systemic approach was used to analyze data obtained from the theoretical and practical bases. Using this method, all stages of the disease were identified, and treatment methods for chronic acute obstructive bronchitis in children were found.

The presented study was conducted at the Institute of Hereditary Pathology of the National Academy of Medical Sciences of Ukraine during 2019–2020. 59 children aged 2 to 8 years with recurrent episodes of acute obstructive bronchitis (I-AOB) and 35 children of the same age who had acute bronchitis (AB) at least 1–2 times a year were examined and compiled as a comparison group (II-ABK). The groups of children had differences in the distribution of age and gender. In two groups of children, there were an almost equal number of boys (58.0%) and girls (42.0%), aged two to eight years. A molecular genetic study was conducted on 35 children with recurrent episodes of acute obstructive bronchitis (I-AOB), and its results were compared with those of 35 children with acute bronchitis (II-ABK).

DNA was isolated from the patient’s peripheral blood leukocytes as the study material. The specificity of the PCR products and the analysis of restriction fragments were performed by electrophoresis in 2–3% agarose gel for 30–40 minutes at a voltage of 100V. The electropherogram was scanned on an ultraviolet Tran’s illuminator. The received signals were compared with length markers and based on this, the sizes of the obtained fragments were detected. According to the requirements of bioethics regarding carrying out laboratory tests of biological material, parents of each child received written consent for biomaterial research.

The results were statistically processed on a personal computer with the calculation of values of the arithmetic mean (M), of the errors arithmetic mean (m), Student criterion (t), the degree of probability (p), Pearson criterion (χ^2^) and the odds ratio (OR). Statistically significant differences were figured at p<0.01 and p<0.05. Student’s criterion (t) is used to test the equality of the mean values in two samples. The Pearson criterion (χ^2^) allows checking the significance of the discrepancy between the empirical (observed) and theoretical (expected) frequencies. The use of the χ2 criterion divides the range of variation of the sample into intervals and determines the number of observations (frequency) for each of the intervals.

## Results

Comparative analysis of the clinical condition of the surveyed children with recurrent episodes of acute obstructive bronchitis and children with acute bronchitis according to clinical, radiological examination, and laboratory parameters is presented in Table 1. The study showed that children with recurrent episodes of acute obstructive bronchitis had a significantly high frequency of hypo oxygenation clinical manifestations: pale skin, reduced saturation, auscultation (such as dry whistling wheezing). Also, they had Percussion changes in the lungs, radiological signs of obstructive bronchitis, eosinophilia, and lymphocytosis in the blood count, compared with data from children with acute bronchitis (Table 1).

**Table 1.** Comparative analysis of the clinical condition of children of 2–8 years with recurrent episodes of acute obstructive bronchitis and the group of children with acute bronchitis.

**Clinical manifestations:**	**Frequency of clinical and laboratory manifestations in groups of children**			
	**I-AOB**		**II-ABK**	
	**n=59**	**q**	**n=35**	**q**
**Pallor of the skin**	39	0.66 *	7	0.20
**Reducing Saturation**	36	0.61 *	–	–
**Auscultatory changes**	54	0.92 *	5	0.14
**Box Percussion Sound**	51	0.86 *	–	–
**X-ray signs of bronchitis**	44	0.75 *	10	0.29
**Leukocytosis**	16	0.27	12	0.34
**Eosinophilia**	31	0.53 *	4	0.11
**Lymphocytosis**	34	0.58 **	9	0.26

* – significant difference between the data rate of children with recurrent episodes of acute obstructive bronchitis and comparison group; p<0.01; ** – significant difference between the data rate of children with recurrent episodes of acute obstructive bronchitis and comparison group; p<0.05; q – Parameter frequency deviation from the norm or the reference value.

The next stage of work performed a molecular genetic study of polymorphic loci C-33T gene *IL-4* in children with recurrent episodes of acute obstructive bronchitis compared with the data of children with acute bronchitis. The results are presented in Table 2.

**Table 2.** Analysis of the distribution of genotypes for polymorphic locus C-33T IL4 gene.

**Genotypes**	**II–AOB, N=35**		**II–ABK, N=35**		**χ^2^**	**p**	**OR, 95% CI**
	**n**	**%**	**n**	**%**			
**CC**	9	26.0 **	18	51.0	4.88	<0.05	0.89 (0.34–2.32)
**CT**	22	63.0 *	10	29.0	8.29	<0.01	4.23 (1.55–11.55)
**TT**	4	11.0	7	20.0	0.97	>0.05	0.52 (0.14–1.95)

* – significant difference between the data rate of children with recurrent acute obstructive bronchitis and comparison group; p<0.01; ** – significant difference between the data rate of children with recurrent episodes of acute obstructive bronchitis and comparison group; p<0.05.

There was a significantly higher rate of genotype 33CT in children with recurrent episodes of acute obstructive bronchitis than children with acute bronchitis (63.0% versus 29.0% in the comparison group, p<0.01). Moreover, there was a significantly higher rate of genotype CC in children with acute bronchitis than children with recurrent episodes of acute obstructive bronchitis (51.0% versus 26.0% in the group of children with recurrent episodes of acute obstructive bronchitis, p<0.05). The study showed that the presence of *IL4* 33CT genotype is associated with susceptibility to repeated episodes of acute obstructive bronchitis and 4-fold increased risk of occurrence (OR 4.23, 95% CI 1.55–11.55).

## Discussion

The persistence of local microbial ecology in monospecies populations within biofilms causes a chronic inflammatory condition beneficial for organisms such as untyped Haemophilus influenzae (NTHi). NTHi uses products such as DNA in neutrophil networks for matrix-building purposes and will benefit from releasing other nutrients into the local environment caused by inflammation [16]. Viral infections promote biofilm formation and induce “flare-ups” described by the release of planktonic organisms. This makes biological sense in that a viral infection will allow further spread within the current host, leading to an “exacerbation” of symptoms outside the natural course of the viral disease [17].

Respiratory diseases are among the most frequent disorders in the clinical practice of every pediatrician. Recurrent viral infections are part of any child’s growing-up process. Especially in children, one can observe some who suffer from recurrent upper or lower respiratory tract infections. In general, a well-off child with recurrent respiratory infections (RRI) does not have a serious illness. Most children do not have immunodeficiency, but if they do, it is often conditioned by an antibody deficiency. With a positive history of immunodeficiency, a detailed immunological examination is necessary. Immunologic testing should be done in other children after other more common causes of RRI have been ruled out, such as gastroesophageal reflux, allergies, or focal ENT infection (adenoid hypertrophy).

Treatment and prevention of these infections have its rules and should include early, targeted antibiotic therapy, acute attacks of infection, long-term and adequate recovery, elimination of all possible foci and sources of infection, and a complete study of the child’s immune status [18]. It is important not to develop a position of treating all obvious viral infections to prevent chronic bronchitis. There are several possibilities for immunomodulatory therapy. Many clinical and experimental studies, including this study, have confirmed their effectiveness and pharmacological safety. The correct appointment and use of each means of immunomodulation should be carried out only in the indicated cases with an individual approach to each child, taking into account all the rules of immunomodulatory therapy. It is equally important to determine the cause of chronically occurring acute bronchitis in children in time using the methods identified in this study.

## Conclusions

Children with recurrent episodes of acute obstructive bronchitis compared to those with acute bronchitis were diagnosed with a significantly higher incidence of clinical manifestations such as pallor, the presence of wheezing, box percussion sound, decreased O_2_ saturation, eosinophilia, and lymphocytosis. It is proved that if a child carries the genotype 33CT of the *IL4* gene, the risk of recurrent episodes of acute obstructive bronchitis increases 4 times. Furthermore, the genotype 33CC in children is a protective factor for repeated episodes of acute obstructive bronchitis.

Further research is required to establish better clinical criteria that may help clinicians diagnose acute obstructive bronchitis. In fact, the proposed criteria for acute obstructive bronchitis are nonspecific and can lead to significant overdiagnosis and overuse of antibiotics, which can cause antibiotic resistance. Further studies are required to identify and treat chronic acute obstructive bronchitis using patient genome analysis.

## Acknowledgements

### Conflict of interest

The authors declare that there is no conflict of interest.

### Ethics approval

The study was approved by the Institute of Hereditary Pathology of the National Academy of Medical Sciences of Ukraine, No. 486/3.

### Consent to participate

Parents of each child gave written consent for biomaterial research.

### Authorship

NL designed the research. OK and ZN performed the research and analyses. DT and MS interpreted data and wrote the manuscript. All authors read and approved the final manuscript.
